# Improvement in Math Ability and Cognitive Processing in Children with Low Attention: An Intervention Based on PASS Theory

**DOI:** 10.3390/jintelligence12090083

**Published:** 2024-08-29

**Authors:** Dan Cai, Yongjing Ge, Lingling Wang, Ada W. S. Leung

**Affiliations:** 1 School of Psychology, Shanghai Normal University, Shanghai 200234, China; caidan@shnu.edu.cn (D.C.); 1000495288@smail.shnu.edu.cn (Y.G.); 2 Huangpu Experimental Primary School Affiliated to Shanghai Jiao Tong University, Shanghai 200020, China; 3 Department of Occupational Therapy, Faculty of Rehabilitation Medicine, University of Alberta, Edmonton, AB T6G 2G4, Canada; awleung@ualberta.ca; 4 Neuroscience and Mental Health Institute, University of Alberta, Edmonton, AB T6G 2E1, Canada

**Keywords:** low attention, math modules, PASS theory, math training

## Abstract

This study investigates the effects of math training on math and cognitive performance among 8–9 year-old students with low attention. Fifty-six students with low attention were randomly assigned to a training group (n = 24) and a passive control group (n = 32). They completed math problem-solving, calculation fluency and PASS cognitive processing tests both before and after training. The children in the training group received 3 days of training per week for a total of 21 days using the math modules of *The Children’s Mathematics and Cognition Training Manual* in Chinese. The results showed that the training group’s math problem-solving performance improved significantly. Moreover, the cognitive performance on the CAS-2 in the planning and simultaneous processing tests for the training group was enhanced. The implications of these findings are discussed with consideration of the interpretability being constrained by the fact that no active control condition was applied.

## 1. Introduction

Attentional resources are necessary for children to initiate and direct their processing of information and comprehend and retrieve information for different tasks (Geary et al. 1999). Studies have shown that low attention may lead to fewer achievements (Mayoral et al. 2021).

Low attention represents lower levels of persistence, task orientation, organization, and focus (Georges et al. 2012). The behaviors of school-aged students with low attention may become a risk factor for poorer classroom behavior and less long-term academic achievement (Salla et al. 2016). For instance, in a 6 year follow-up study on preschool children (Sayal et al. 2020), it was found that baseline inattention was associated with poorer academic outcomes.

The term “low attention” was used to describe students with scores below their grade levels for attention assessment which were not explained by a formal diagnosis of attention deficit hyperactivity disorder (ADHD). These students received the same education as typical developing students. Although they did not have any serious behavioral problems like ADHD, they performed much worse in tasks requiring attention in comparison with typical children.

### 1.1. Math Module Training Program

Children must grasp certain logical principles in order to understand mathematics (Piaget 1976). By mid-elementary school, some children have completely lost interest in learning math, mostly because they experienced great difficulties while learning mathematical concepts. Recently, scientific research has introduced various rules and tools to help children learn mathematics. Modules for Math, a manual for math training, is an intervention program which uses cognitive training techniques to target the basic skills needed for mathematical proficiency (Cai et al. 2015; Das 2020). Modules for Math targets basic math skills and includes training programs which are expected to improve them. The basic skills are general cognitive processes which lead to the development and learning of mathematical facts such as addition, subtraction, multiplication and division.

**Figure 1:**
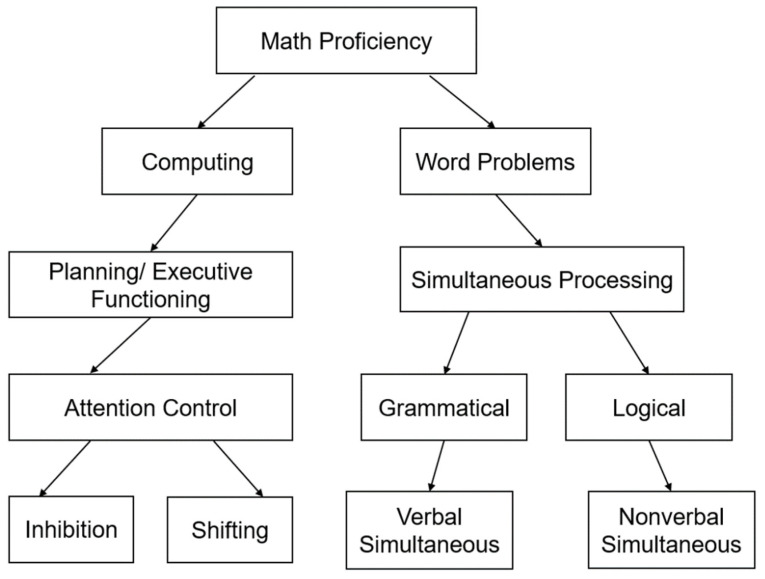
Math proficiency model.

Figure 1 below outlines the components of math proficiency, which is the collective aim of Modules for Math. It shows the familiar division of math proficiency into computing and word problems. Planning and executive functioning (EF) is the predominant cognitive process for computing. Similarly, simultaneous processing is required for the comprehension of word problems. Attentional control (Geary 2013), as proposed, is subsumed in planning and EF, whereas simultaneous processing comprises logical grammatical relations (following (Luria 1966) in PASS theory). The last level highlights the two components of EF: inhibition and shifting. For word problems, logical and grammatical divisions are to be measured by non-verbal (matrix-type tests) and verbal simultaneous tasks.

**Figure 2:**
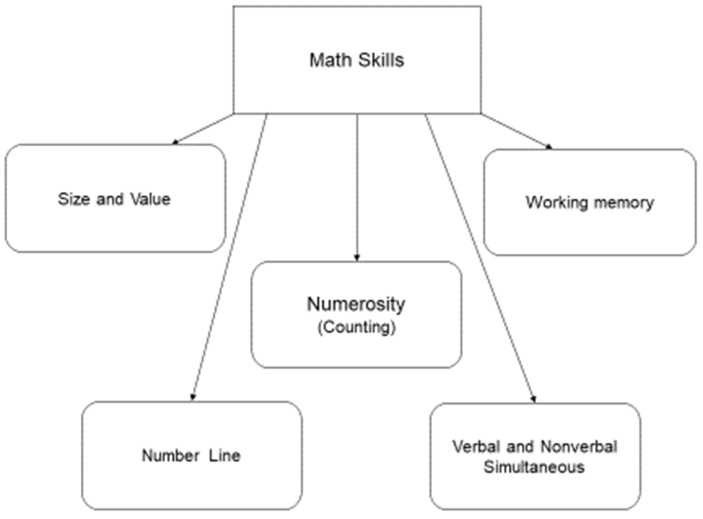
The five basic math skills.

**Figure 3:**
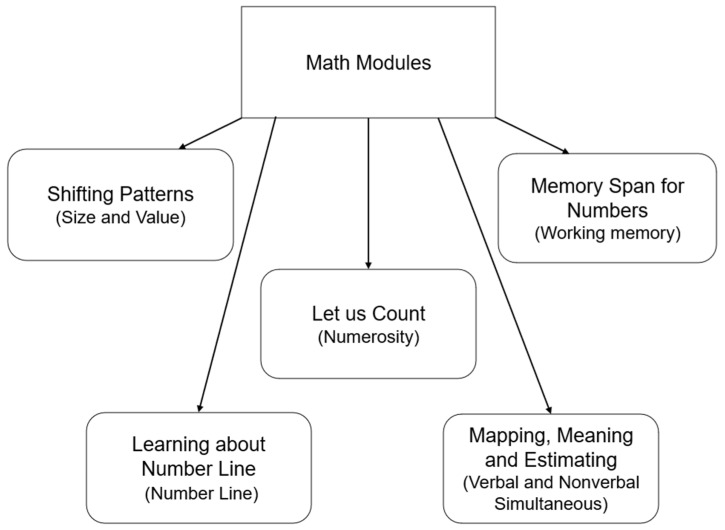
The five modules of Modules for Math.

Modules for Math selected five math skills which are most frequently mentioned in the current literature and academia: size and value, number line, numerosity (counting), verbal and non-verbal simultaneous, and working memory. Therefore, the manual set five modules, and each module’s objective was one of the math skills (see Figure 2 and Figure 3).

*The Children’s Mathematics and Cognition Training Manual* in Chinese (Das and Cai 2017) was formulated based on Modules for Math and PASS theory to effectively evaluate and help children with different aspects of cognitive and academic difficulties (Cai et al. 2015). The training program aims to improve PASS cognitive processing abilities through a series of intervention activities, which includes five modules based on the four processes of PASS, namely shifting, number line, mapping and estimating, counting, and memory span for number. Cai et al. (2015) concluded that this battery might help improve students’ mathematical abilities.

### 1.2. Assessment of Cognitive Processes

Planning, attention, simultaneous, and successive (PASS) theory proposes that cognition is composed of three systems and four cognitive processes (Das et al. 1994). The first system is planning, which involves the executive functions responsible for organizing and supervising behavior. The second system is attention, which is responsible for maintaining arousal levels such that individuals can focus on relevant stimuli. The third system is simultaneous and successive processing. Simultaneous processing is required to analyze and synthesize the logical grammatical relationships in both verbal and non-verbal problems. Successive processing is required to organize separate items sequentially, such as remembering a sequence of words or actions exactly in the order in which they were presented. Naglieri and Das (1997) developed a set of standardized tests based on PASS theory, namely the Das–Naglieri cognitive assessment system (CAS), which proposed a series of tasks to measure the four cognitive processes of PASS theory. This individually administered test of neurocognitive functioning was standardized for use with children and adolescents ranging from 5 to 17 years of age (Georgiou et al. 2021). The CAS predicts students’ performance in cognitive processing tasks (Naglieri and Das 1997; Naglieri and Rojahn 2004). Some studies have provided evidence that the CAS is reliable in different languages (for English, see Das et al. (2008); for Chinese, see Wang et al. (2012)) and different age groups (Naglieri and Rojahn 2004).

Previous studies have shown that PASS cognitive processes are related to different types of mathematical performance (Das and Janzen 2004; Kroesbergen et al. 2003; Naglieri and Das 1997). For example, Naglieri and Das found that planning was required in making decisions about how to solve a math problem, while simultaneous processing was particularly important when integrating different and interrelated elements into a whole to answer math problems. In particular, Cai et al. (2013) concluded that students with mathematical learning difficulties (MLDs) performed worse than a control group in all PASS processes.

### 1.3. Cognitive Intervention Programs

At present, several studies have focused on cognitive intervention, showing that different types of cognitive interventions have different effects on students’ cognitive and academic abilities (Banales et al. 2015; Olesen et al. 2004; Peng and Miller 2016). For example, attentional control in ADHD students (Gaál and Czigler 2018; García-Baos et al. 2019), executive function (Steiner et al. 2011) and working memory (Klingberg et al. 2002; Tamm et al. 2010) have been significantly improved after cognitive intervention. All of these “intervention studies” are to be validated by transferring training to general tasks which are not part of the specific training tasks (i.e., far transfer). However, one meta-analysis study did not show evidence to support far transfer to nonverbal ability, verbal ability or word decoding (Melby-Lervåg et al. 2016). Therefore, the current study will not only examine near transfer to math achievement but also far transfer to underlying cognitive performance in the cognitive processes of PASS cognitive processes.

### 1.4. The Purpose of the Present Study

Based on what has been discussed above, it should be noted that the intervention effect of the cognitive intervention math module in students with low attention remains under-researched, despite the strong association between attention and math performance. It is of great importance to fill research gaps, which could help with designing targeted cognitive intervention programs based on PASS cognitive processes to improve the math and cognitive performance of students with low attention. Therefore, the current research used *The Children’s Mathematics and Cognition Training Manual* to examine the efficacy of math intervention for students with low attention. We expected that the math module training program would lead to near transfer to math achievement. However, given that the examination of the far transfer effect was exploratory in nature, we did not propose a specific hypothesis regarding whether or not the far transfer effect would be found.

## 2. Material and Methods

### 2.1. Participants

We used four attention tests to screen students with low attention, which included the trail making number, receptive attention, number correct and trail making figure, among 576 students from grade one to grade four in a primary school in Shanghai. We defined students with low attention as those whose mean value of the z score of these four tasks was lower than 20% (Cai et al. 2010). A total of 90 students with low attention were eligible to participate in this study. Among them, the parents of 59 students signed informed consent forms.

Fifty-nine students were randomly allocated to the intervention group (N = 27) and the control group (N = 32). Three students in the intervention group dropped out of this study during the intervention. Finally, 24 students in the intervention group (7 girls; mean age = 95.75 ± 8.85 months, ranging from 82 to 112 months) and 32 students in the control group (12 girls; mean age = 98.53 ± 11.04 months, ranging from 81 to 115 months) were included in this study. The z scores of the attention tests in these students ranged from −2.68 to −1.09. There were no significant differences in age or gender between the two groups (F = 1.03, *p* = 0.32, Chi square = 0.43, *p* = 0.52). The teachers reported that all of the participants were Mandarin speakers and right-handed, with no intellectual, sensory or behavioral disabilities.

### 2.2. Measures

#### 2.2.1. Tests for Screening Students with Low Attention

The trail making number was revised by Diller et al. (1974) according to the cancellation test. Students were asked to quickly and accurately mark the number “9” with two lines within a specified time, but if the number “9” with two lines was preceded by the number “5”, then they should not have marked it. The task included three pages, and each page had a 60 s time limit for completion. The number correct (total number minus the number of false detections) for each page was recorded. The Cronbach’s alpha value for the current sample was 0.89. We suggest this test as an index of attention and inhibition.

Receptive attention is a four-page subtest within the CAS-2 (Naglieri et al. 2014), with the first two pages allowing a 60 s time limit and the last two pages allowing a 120 s time limit for completion. In the first two pages, students were asked to identify as quickly as possible the target pairs of letters among distractors (e.g., AA but not Aa). In the last two pages, students were asked to identify letters with the same name (e.g., Aa but not Ba). The number correct (total number of correct answers minus false detections) for each page was recorded. The Cronbach’s alpha value for the current sample was 0.71. It is important to note that the text of the tests used from the CAS-2, like in the Das–Naglieri CAS, were unaltered, except for instruction in Chinese during administration to assist Chinese students with English words.

The number detection task was used to measure attention. The task consisted of four pages of numbers which were printed in different formats. On each page, students were required to find a particular stimulus (e.g., the numbers 1 and 2 printed in an open font) among distractors (e.g., the same numbers printed in a different font). Students were given a 150 s time limit to complete each page. The Cronbach’s alpha value for the current sample was 0.83.

The trail making figure was selected from the Test of Everyday Attention for Children, Second Edition (TEA-Ch2) (Manly et al. 2001). The students were asked to identify the target stimulus in a set of 32 matrix stimuli—a yellow oval—and draw a line on it. The task consisted of two pages, each with a 30 s time limit for completion. The number correct (total number of correct answers minus false detections) for each page was recorded. The Cronbach’s alpha value for the current sample was 0.83.

#### 2.2.2. Math Achievement Tests

Two measures were administered to assess math achievement.

Math problem-solving was selected from the Wechsler Individual Achievement Test, Third Edition (WIAT-III; (Wechsler 2009)). The test consisted of 72 items. The number of math problems correctly solved was recorded. The test was discontinued if a student gave four consecutive incorrect responses. The Cronbach’s alpha reliability coefficient in the previous study was 0.90 (Cai et al. 2018).

Calculation fluency was selected from the Wechsler Individual Achievement Test, Third Edition (WIAT-III; (Wechsler 2009)), which includes three subtests: addition fluency, subtraction fluency and multiplication fluency. In each subtest, students were asked to solve as many items as possible within a 60 s time limit. Each subtest included two pages. Each student’s score was the total number of additions, subtractions and multiplications correctly completed within the time limit. The Cronbach’s alpha values for the addition, subtraction and multiplication subtests for the current sample were 0.94, 0.93 and 0.94, respectively.

#### 2.2.3. PASS Cognitive Processing Tests

The PASS cognitive processes were measured using the CAS-2 (Naglieri et al. 2014), which was organized into four aspects (planning, attention, simultaneous and successive) according to PASS theory. In this study, we used three aspects (planning, simultaneous and successive) as test measures, given that the attention measures were used as screening measures.

Planning was measured with the planned codes task and the matching number task. Students were required to fill in empty boxes in different arrangements according to a legend at the top of each page as quickly as possible. The legends showed which letters corresponded to specified codes. The students were given a 60 s time limit to fill in as many empty boxes as possible using any strategies. The time and number correct for each page were recorded. The Cronbach’s alpha value for the current sample was 0.62. In the matching numbers task, the students were presented with three pages, each with eight rows of numbers. For each row, they were instructed to underline two identical numbers as quickly as possible. The first two pages had a 150 s time limit, and the last page had a 180 s time limit for completion. The time and number of correct matches for each page were recorded. The Cronbach’s alpha value for the current sample was 0.64.

For simultaneous processing the nonverbal matrices task was used to measure simultaneous processing. The task consisted of 44 questions, each presented with shapes and geometric designs which were spatially and logically interrelated within a visual matrix left with a missing space. For each question, the students were required to decode the relationships of the visual matrix and choose from a list of six options to occupy the missing space in the grid. The task was discontinued after four consecutive errors. The number of correct answers was recorded. The Cronbach’s alpha reliability coefficient in the previous study was 0.91 (Naglieri et al. 2014).

To assess successive processing, the digit forward task and word series task were used. In the digit forward task, the examiner read a series of numbers, varying in length from two to nine numbers, presented at a rate of one word per second. The students were required to repeat the numbers in the same order. A discontinuation rule of four consecutive mistakes was applied. Each student’s score was the total number of number series correctly repeated. In the word series task, students were required to repeat a series of Chinese single-syllable words in the same order as that stated by the examiner. Each series ranged in length from two to nine words. A discontinuation rule of four consecutive mistakes was applied. Each student’s score was the total number of number series correctly repeated. The Cronbach’s alpha value of successive processing for the current sample was 0.66.

### 2.3. Intervention Sessions

The cognitive intervention material was selected from *The Children’s Mathematics and Cognition Training Manual* (Das and Cai 2017), which included five modules: shifting patterns, number line, counting; mapping and estimating and memory span for number. Diagrams of the selected modules and the skills for each diagram are shown in Figure 1, Figure 2 and Figure 3.

Module 1: shifting patterns. Students were required to draw a line to connect a series of shapes in the correct sequence within a box from smallest to largest. The connected graphs made by the students could be in the shape of a “Z” pattern or an “N” pattern. This task also required the students to shift their responses from the Z to N pattern or from the N to Z pattern.

Module 2: number line. Students were presented with many pairs of numbers varying in size one at a time. The students had to tell whether the second number was bigger or smaller than the first one. The task was divided into four conditions: neutrality, congruency, incongruency and opposite. In the neutral condition, the numbers were kept at the same printing size, and the students concentrated only on the differences in the actual numbers (e.g., 20 and 31). In the congruency condition, the numbers and sizes were made congruent such that the size of a bigger number was bigger than the size of a smaller number (e.g., 32 and 20). In the incongruency condition, the numbers and sizes were made incongruent, meaning that the size of a bigger number was smaller than the size of a smaller number (e.g., 21 and 40). Students had to observe the numbers carefully before responding “big” or “small”. In the opposite condition, the patterns were reversed. The numbers were the same printing size, but students needed to give the opposite response; that is, they had to say smaller if the second number was bigger than the first number and bigger if the second number was smaller than the first number.

Module 3: counting. Students were presented with a series of boxes containing either three or seven digits, where the digits were either sevens or threes. The task required the students to count the digits from one box to another until they reached the end of the page. The task was divided into two conditions. In the congruency condition, the numbers were congruent in size (e.g., 333 and 7777777). In the incongruency condition, the numbers were incongruent in size, meaning that the number with a smaller value was greater in size compared with the number with a greater value (e.g., 777 and 3333333).

Module 4: mapping and estimating. The first part was the verbal spatial task, which required the students to hear a sentence (e.g., “An arrow pointing to a circle containing three squares.”) and draw different combinations of figures according to the sentences they heard. The task was discontinued if a student gave four consecutive incorrect responses. The second part was the non-symbol estimation task. Students were presented with two boxes on each page, and each box contained a different number of black dots. The students were required to estimate which box had more dots without needing to count them within 1–3 s accurately.

Module 5: memory span for number. Students were asked to listen to a group of simple addition equations and tell quickly whether each addition equation was correct while memorizing the last number of the addition equation. For example, the administrator guided the students by asking, “Is 2 + 3 = 5 true?” The students responded with either “yes” or “no” while memorizing the last number “5”. There were essentially two levels of difficulty. The beginner level involved equations with two numbers (e.g., 2 + 3 = 5). The intermediate level involved equations with three numbers (e.g., 4 + 3 + 1 = 8). The span increased from 2 (two equations) to 7 (seven equations). After all of the equations were presented, the students had to report the last numbers of each equation in the same order in which they were presented.

### 2.4. Procedures

The attention tests took place in classrooms at the students’ schools during school hours. Three primary teachers and 16 graduate students who were trained rigorously conducted the testing. Data collection included one 45 min classroom session (for attention tests) and one 40 min individual session (for all other pre- and post-training outcome testing). After screening the low-attention students, they were randomly allocated into an intervention group or a control group.

### 2.5. Intervention Process

The intervention was conducted online at home over a period of 7 weeks, with 3 days of intervention per week for a total of 21 days of intervention (each session lasting 30–40 min). Before the intervention day, the parents were given intervention sessions, guidance, intervention demonstration videos and intervention materials, including log sheets and feedback forms, from the experimenter via the internet. Every Saturday, the experimenter collected the feedback forms for that week’s intervention from the parents. Students from the control group were given regular school activities online as usual.

### 2.6. Testing Process

The PASS cognitive processing ability and math ability tests were conducted before (pre-test) and after (post-test) intervention in a quiet room at school during school hours. Testing lasted about 40 min. Graduate students conducted the testing for 56 students in the intervention group and control group separately, and they were blinded to the group membership of the study. After completing testing, each student received a small gift.

### 2.7. Data Analysis

The intervention’s effects and outcome measures (math achievement and PASS cognitive tests) were examined using 2 × 2 repeated measures ANOVA, with time (pre-test and post-test) as the within-subject factor and group (the intervention group and the control group) as the between-subject factor. Statistical significance was set at *p* < 0.05, and all statistical analysis was conducted on SPSS 21.0. Bonferroni correction was applied to adjust for multiple comparisons in the analysis.

## 3. Results

The pre-test and post-test means and standard deviations of the outcome measures in each group are presented in Table 1.

**Figure 4:**
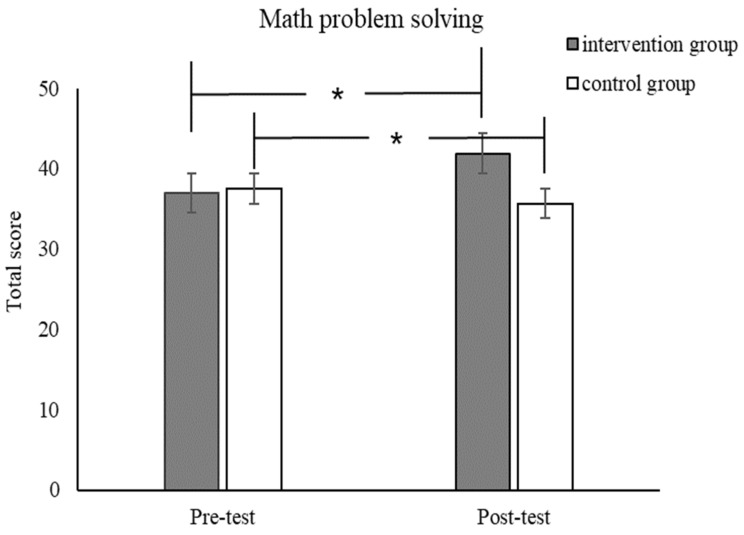
Pre-test and post-test scores for math problem-solving for the intervention and control groups. * *p* < 0.05.

For the near transfer effect, we found a significant main effect from the group (*F*_(1,53)_ = 13.11, *p* < 0.001, *p_Bonferroni_* < 0.01, *η_p_*^2^ = 0.20) for math problem-solving. More importantly, there was a significant interaction effect from the time and group in math problem-solving (*F*_(1,53)_ = 28.19, *p* < 0.001, *p_Bonferroni_* < 0.01, *η_p_*^2^ = 0.35, Figure 4).

**Figure 5:**
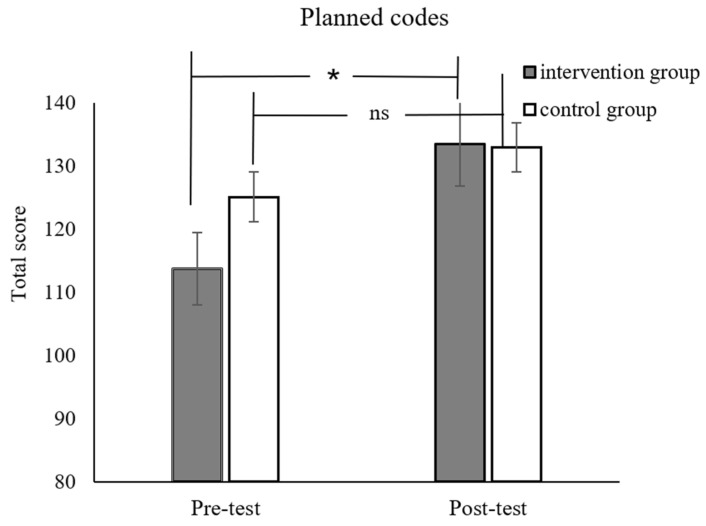
Pre-test and post-test scores for planned codes for the intervention and control groups. * *p* < 0.05; ns = nonsignificant.

**Figure 6:**
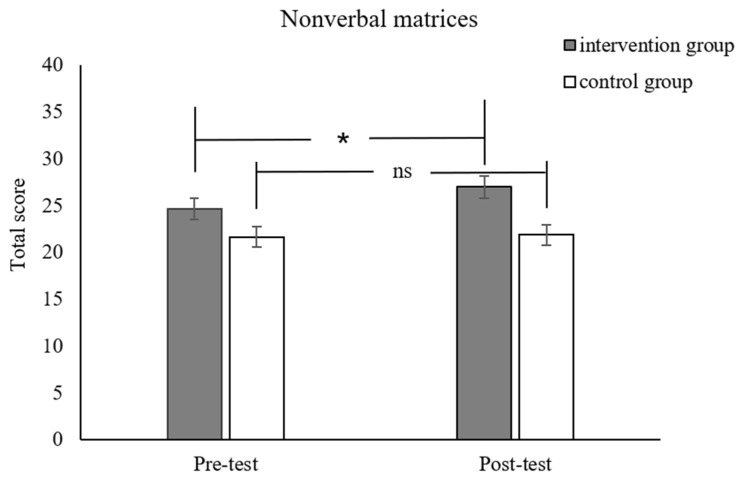
Pre-test and post-test scores for nonverbal matrices for the intervention and control groups. * *p* < 0.05; ns = nonsignificant.

For the far transfer effect, the interaction effects of the time and group for planned codes (*F*_(1,53)_ = 5.37, *p* = 0.02, *p_Bonferroni_* = .14, *η_p_*^2^ = 0.09, Figure 5) and nonverbal matrices were in the right direction (*F*_(1,53)_ = 3.27, *p* = 0.07, *p_Bonferroni_* = .49, *η_p_*^2^ = 0.09, Figure 6). However, it should be noted that these results did not survive the Bonferroni correction. Given that this was an exploration, we went on to conduct simple effect analysis on these results. It revealed that the post-test performance of the intervention group was better than its pre-test performance. For the control group, there were no significant differences pre- and post-test. There was no significant difference between the two groups in the arithmetic fluency, matching number, digit forward and word series tasks. Detailed results can be seen in Table 2.

Moreover, we also conducted additional analysis on the gain score (post-test performance minus the pre-test performance) for each group. The results indicated that the experimental group showed significantly higher gain scores in the math problem-solving tasks (*t*_54_ = 5.50, *p* < 0.001, *p_Bonferroni_* < 0.01). The group difference in the planned codes task (*t*_54_ = 2.10, *p* = 0.04, *p_Bonferroni_* = 0.28) and nonverbal matrices task (*t*_54_ = 1.18, *p* = 0.07, *p_Bonferroni_* = 0.49) was in the right direction again, but it did not survive the Bonferroni correction.

## 4. Discussion

The present study examined the effects of math intervention on academic skills and various types of cognitive performance. We used *The Children’s Mathematics and Cognition Training Manual* (Das and Cai 2017) to examine the math intervention effects for students with low attention. As hypothesized, the results showed that the performance of low-attention students in math problem-solving improved after the math interventions. The results also showed that planning and simultaneous processing of the intervention group changed in the right direction. However, it should be noted that these two results did not survive the Bonferroni correction, warranting further investigation. Moreover, we have to acknowledge that no active control group was included. Although a recent study with aggregated data from 1524 cognitive training studies demonstrated no evidence that a passive or active control group type meaningfully influences the effect sizes of the objective cognitive measures (Au et al. 2020), a study without an active control group which has the same expectation of improvement as the experimental group does not permit the inference of any specific conclusion regarding the effectiveness of the training (Boot et al. 2013).

The results were partly consistent with previous studies. For example, Alloway (2012) found improvements in math ability after 25 sessions of cognitive training. Furthermore, as reported by Zhang et al. (2019), math problem-solving is considered a high-level math skill which involves several complex cognitive processes, such as planning and nonverbal cognitive ability. Previous research also supported these attentional abilities playing a vital role in mathematics skills. For example, Geary (2013) found that students’ attentional abilities were the basis of mathematics skills, even after accounting for general intelligence (Blair and Razza 2007). Children who score high in attention tasks may understand how to count objects better than those who score low in attention tasks, enabling them to allocate more attentional resources to perform complex tasks, such as math problem-solving (Gersten and Chard 1999).

A previous meta-analysis study found no evidence of far transfer (Melby-Lervåg et al. 2016). However, we reported a significant far transfer effect here. One explanation why we found far transfer effects from training low-attention students may be that all tasks involve a global training component and an additional curriculum-related bridging component in the current intervention program. The global component consists of structured tasks which require the application of key cognitive strategies. For math modules, strategies comprise planning and executive functions and simultaneous processing. Therefore, the tasks in *The Children’s Mathematics and Cognition Training Manual* allow children to internalize strategies in their own way, thus facilitating transfer (Das 2020). The bridging component involves the same cognitive demands as its global component. The global and bridging components are further divided into levels of difficulty. This allows the students to progress in strategy development and, for those who already have some successful processing strategies in place, begin at an appropriate level of difficulty. Furthermore, a system of prompts is also integrated into each global and bridging component. The prompts support and guide the child to ensure that he or she completes the tasks with minimal assistance and maximal success. The training program is constructed within a theoretical framework supported by Vygotsky (zones of proximal development) (Vygotsky and Cole 1978) and Luria’s self-regulating role of language, which emphasizes the transition from external speech to internalization (Luria 1966).

Transfer of learning is at the core of improvement following cognitive training. Two kinds of transfer have been proposed in educational psychology. “Low-road transfer depends on extensive, varied practice and occurs by the automatic triggering of well-learned behavior in a new context. High-road transfer occurs by intentional mindful abstraction of something from one context and application in a new context” (Salomon and Perkins 1989). The kind of transfer we have claimed to be successful, as discussed below, certainly qualifies as “high road”, but with a certain difference: It may be implicit rather than intentional, mindful abstraction. In fact, when asked, “Tell me how you did it”, all students may not be able to give an answer. This knowledge may not be accessible to consciousness.

Another explanation of the far transfer effect we found may be due to self-talk or verbalization, which is recommended as a technique for organizing strategies while solving a math problem (Papadopoulos et al. 2015). We suggest verbalization as a mediating procedure which not only enhances local operations such as multiplication and division but also facilitates the transfer of training to improvements in basic math skills. In this manner, children could strengthen their use of planning, self-reflection, verbalizing the methods employed and self-evaluation (Iseman and Naglieri 2011).

It should be noted that this study has some limitations. As mentioned before, the biggest pitfall of the current study is the passive control group used. A much more ideal research regime would use other tasks which are also cognitively demanding and perceived as effective training but lack the math component. Our sample was relatively small. This small sample size may be the reason why we found no evidence of post-test gain for the planned codes task in the control group. Therefore, it is acknowledged that future research should focus on expanding the sample size and increasing the age range and school grade range of students. Second, we did not examine the long-term efficacy of the math intervention program. Moreover, we did not recruit an active control group. Low attention frequently co-occurs with other non-behavioral problem traits such as stress (Whiting et al. 2021) and anxiety (Justicia-Galiano et al. 2017), which could exert a significant influence on the reported study results. We used the same attention tasks to identify students with low attention and measure their pre- and post-intervention ability. This may confound the present results. Future studies are encouraged to use different tasks in order to avoid such confounding factors.

As this was the first study which explored the effect of math intervention based on math modules in low-attention students, the results need to be replicated with an active control condition before strong conclusions can be drawn. Overall, the current preliminary findings provide a small hint that intervention based on math modules might have effects on not only the math skills but also the underlying cognitive performance in planning and attention in low-attention students.

## Figures and Tables

**Table 1:** Means and standard deviations (SDs) of performance in each task for intervention and control groups.

	Intervention Group (*N* = 24)		Control Group (*N* = 32)	
	Pre-Test	Post-Test	Pre-Test	Post-Test
**Math Achievement**				
Math Problem Solving	36.96 (7.32)	41.88 (5.47)	37.52 (5.26)	35.65 (4.85)
Arithmetic Fluency	56.04 (23.79)	70.96 (20.56)	54.09 (24.55)	68.24 (23.67)
**Executive Functions (Planning)**				
Planned Codes	113.77 (32.97)	133.50 (30.99)	125.16 (27.69)	132.96 (33.59)
Matching Numbers	16.92 (3.45)	15.50 (2.98)	16.74 (3.89)	14.94 (3.18)
**Simultaneous Processing**				
Nonverbal Matrices	24.69 (4.68)	27.00 (5.77)	21.65 (3.74)	21.89 (3.65)
**Successive Processing**				
Digit Forward	24.20 (3.68)	23.88 (3.89)	22.43 (4.48)	22.45 (4.32)
Word Series	19.69 (2.73)	21.42 (4.30)	19.51 (1.68)	20.50 (3.98)

**Table 2:** Repeated measures ANOVA results.

Task	Time			Group			Time × Group		
	*F*	*p*	*η_p_* ^2^	*F*	*p*	*η_p_* ^2^	*F*	*p*	*η_p_* ^2^
**Math Achievement**									
Math Problem Solving	4.03	0.05 *	0.07	13.11	<0.001 **	0.20	28.19	<0.001 **	0.35
Arithmetic Fluency	1.34	0.25	0.03	3.08	0.09	0.06	0.02	0.88	<0.001
**Executive Functions (Planning)**									
Planned Codes	1.06	0.31	<0.001	.00	0.99	0.01	5.37	0.02 *	0.09
Matching Numbers	4.38	0.04 *	0.08	.63	0.43	0.01	<0.001	0.96	<0.001
**Simultaneous Processing**									
Nonverbal Matrices	0.27	0.60	0.01	16.20	<0.001 **	0.23	3.27	0.07	0.06
**Successive Processing**									
Digit Forward	0.40	0.53	0.01	2.79	0.10	0.05	0.11	0.74	<0.001
Word Series	2.96	0.09	0.05	0.75	0.39	0.01	0.90	0.35	0.02

Note. * = *p* < 0.05; ** = *p* < 0.01.

## Data Availability

The data will be made available on request from the corresponding author.
